# Correction: Plasmids manipulate bacterial behaviour through translational regulatory crosstalk

**DOI:** 10.1371/journal.pbio.3002531

**Published:** 2024-02-15

**Authors:** Catriona M. A. Thompson, James P. J. Hall, Govind Chandra, Carlo Martins, Gerhard Saalbach, Supakan Panturat, Susannah M. Bird, Samuel Ford, Richard H. Little, Ainelen Piazza, Ellie Harrison, Robert W. Jackson, Michael A. Brockhurst, Jacob G. Malone

The graph in Fig 8A was affected by a transposition error prior to publication. The authors have provided a corrected version here.

In the second paragraph of the Results sub-section ‘Carbon source sensing by RsmQ,’ the sixth sentence is incorrect. The correct sentence reads: Interestingly, cells carrying pQBR103Km –ΔrsmQ are able to metabolise citric acid and D-sorbitol at similar levels to WT cells, suggesting that RsmQ is able to repress metabolism of these carbon sources.

The third paragraph in the Results sub-section ‘Carbon source sensing by RsmQ’ should be disregarded.

**Fig 8:**
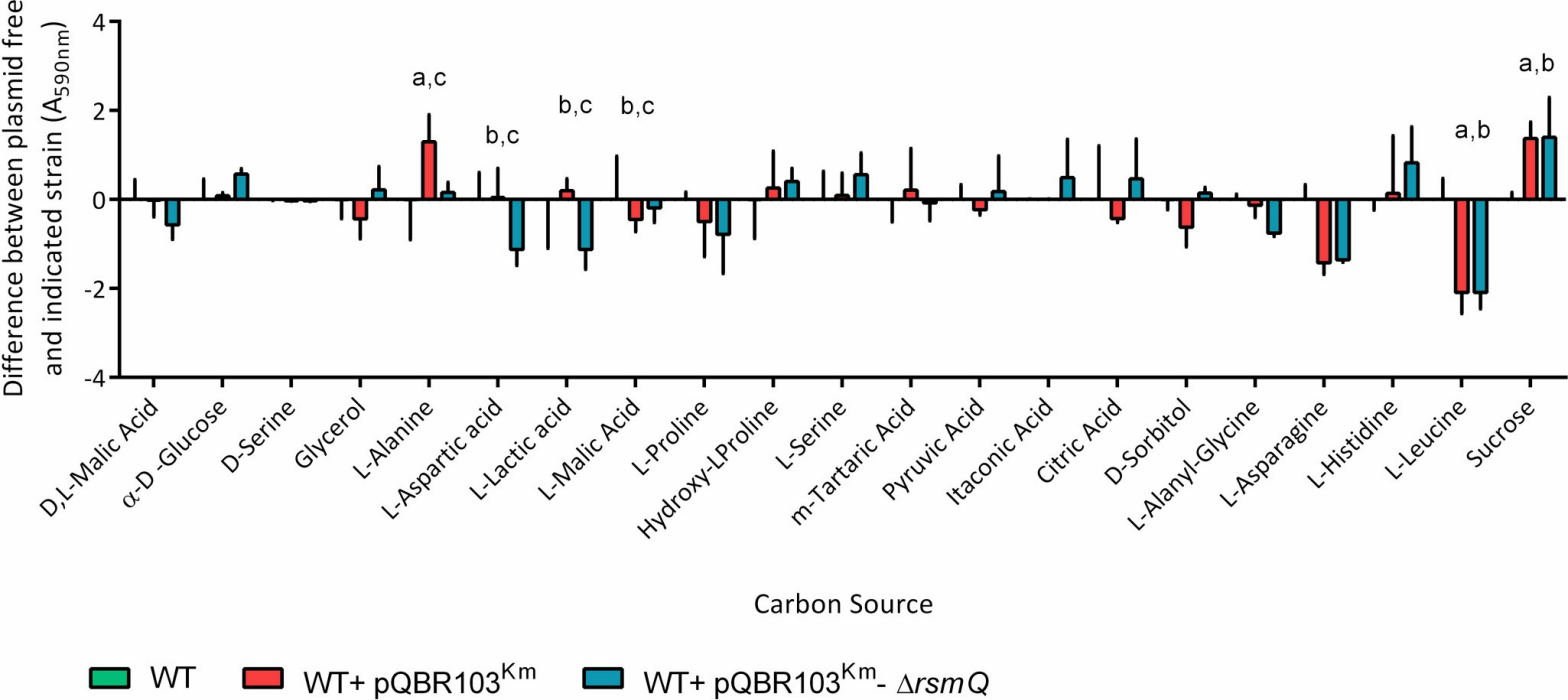
RsmQ is involved in the control of carbon source metabolism. (a) Selected results from BioLog carbon source screens showing metabolism with relevant carbon sources after 48 h of static incubation at 28°C. Data are shown for SBW25 cells carrying pQBR103Km (red) and pQBR103Km-ΔrsmQ (blue) compared to average SBW25 plasmid free (green) for each carbon source. Error bars show standard deviation. A two-way ANOVA showed a significant effect of carbon source (p < 0.0001) as well as a significant interaction (p < 0.0001), but no effects of plasmid in this chosen dataset. Multiple comparisons compared SBW25 plasmid free, SBW25 +pQBR103Km and SBW25 +pQBR103Km- ΔrsmQ within each carbon source. Significant differences are indicated above for SBW25/SBW25 +pQBR103Km (a), SBW25/SBW25 +pQBR103Km-ΔrsmQ (b), and SBW25 +pQBR103Km /SBW25 +pQBR103Km-ΔrsmQ (c) for p < 0.05. Data for all carbon sources can be seen in S11 Fig and S2 Table. (b) Growth curves are shown for SBW25 (green), SBW25 cells carrying pQBR103Km (red), and pQBR103Km-ΔrsmQ (blue), with the mean growth for 3 biological replicates shown as a solid line and standard deviation shown as dotted lines. Cells were grown for 48 h at 28°C without shaking in M9 minimal media with 0.4% w/v either D-sorbitol, glycerol, L-alanine, citric acid, L-aspartic acid, L-histidine as indicated. Data are available in S7 Data. WT, wild-type.

## References

[1] Thompson CMA, Hall JPJ, Chandra G, Martins C, Saalbach G, Panturat S, et al. (2023) Plasmids manipulate bacterial behaviour through translational regulatory crosstalk. PLoS Biol 21(2): e3001988. doi: 10.1371/journal.pbio.3001988 36787297 PMC9928087

