# Supplementary figures and images for: Plasmids manipulate bacterial behaviour through translational regulatory crosstalk
Source: PLoS Biol. 2023 Feb 14;21(2):e3001988. doi: 10.1371/journal.pbio.3001988 (PMC9928087; doi:10.1371/journal.pbio.3001988)

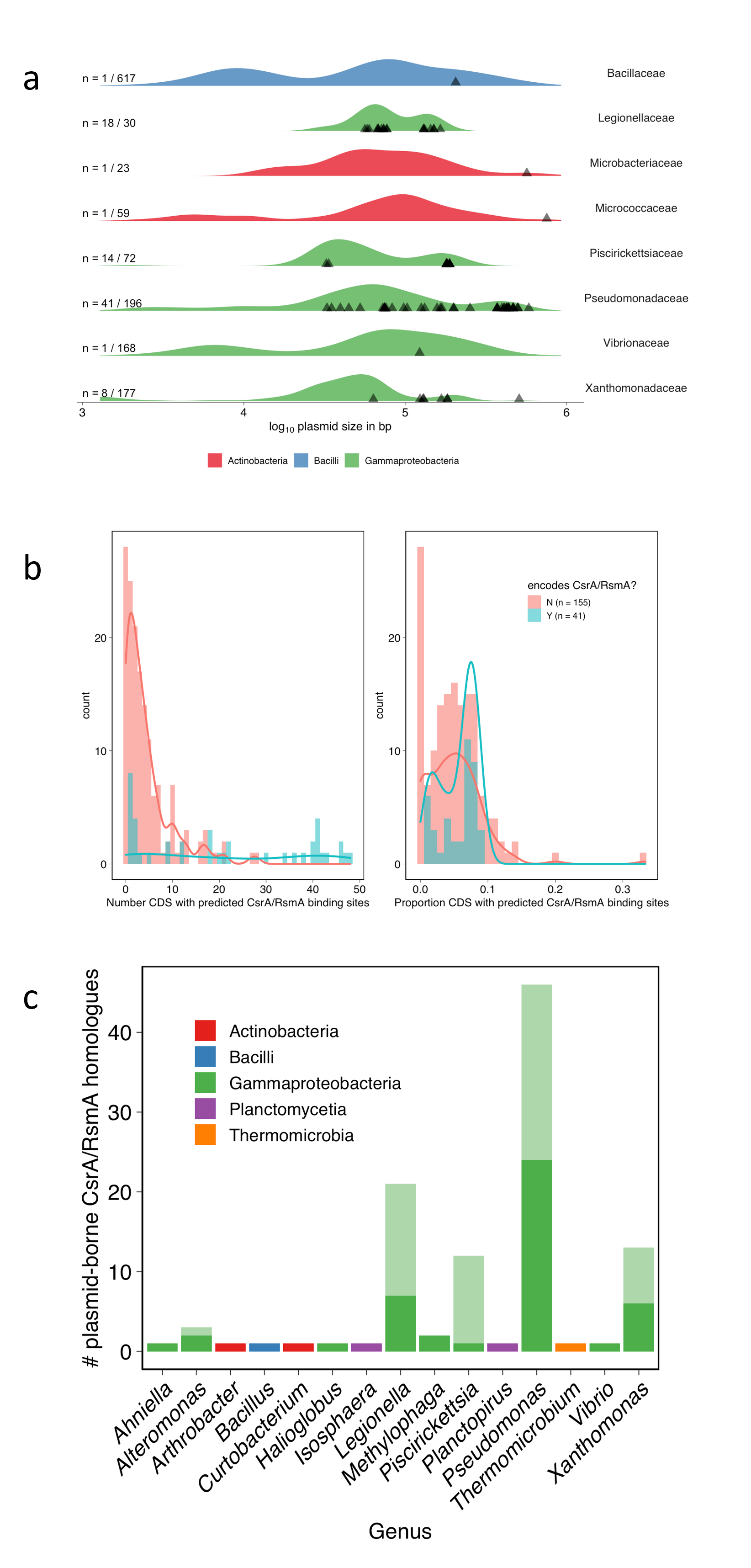

Supplement: S1 Fig — (a) Across Families, CsrA/RsmA-encoding plasmids are relatively large. Size density plots for all Families with >20 plasmids and ≥1 plasmid-encoded CsrA/RsmA homologue. Each row describes a different Family. Semi-transparent triangles indicate the size of CsrA/RsmA-encoding plasmids. On the left, the proportion of total plasmids encoding CsrA/RsmA homologues for that Family. (b) Comparison of putative CsrA/RsmA-regulated gene frequencies between CsrA/RsmA-encoding and non-encoding Pseudomonadaceae plasmids. Plots show overlayed histograms and density plots. Left-hand plot shows absolute numbers of putative CsrA/RsmA-regulated genes, whereas right-hand plot shows as a proportion of total CDS on that plasmid. Distributions were significantly different between plasmid types in both panels (Kolmogorov–Smirnov test, p < 0.001 for absolute counts, p = 0.012 for proportions). (c) Taxonomic distribution of plasmid borne csrA/rsmA homologues identified in COMPASS as in Fig 1. The paler part of each stacked bar indicates genes that were identical at the nucleotide level to other identified homologues. All data and analyses are available on github (PLASMAN_RsmQ) with data taken from the COMPASS database. (TIF) [file pbio.3001988.s001.tif]

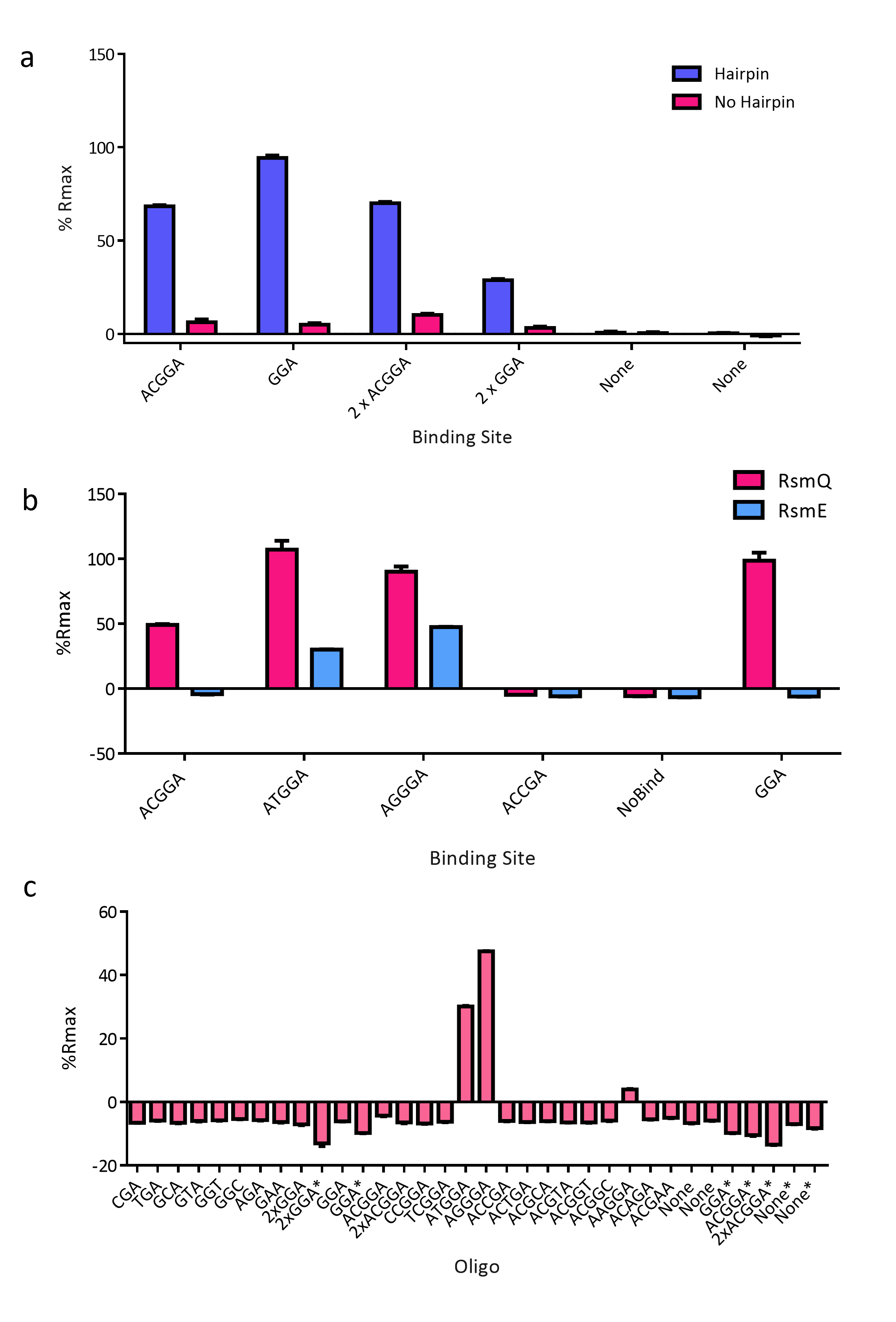

Supplement: S2 Fig — (a) Percentage Rmax values for RsmQ binding to ssDNAs containing the indicated binding site sequence in either a linear format (pink) or at the top of a hairpin loop (purple). (b) Percentage Rmax values for RsmE binding to a selection of ssDNAs compared to RsmQ for selected key oligos. All assays were performed in duplicate and data for 100 mM shown. (c) Percentage Rmax values for RsmE binding to all synthesised oligos with non-hairpin oligos indicated (*). Error bars on all graphs show standard deviation of 2 independent replicates at a concentration of 100 nM. Data are available in S8 Data. (TIF) [file pbio.3001988.s002.tif]

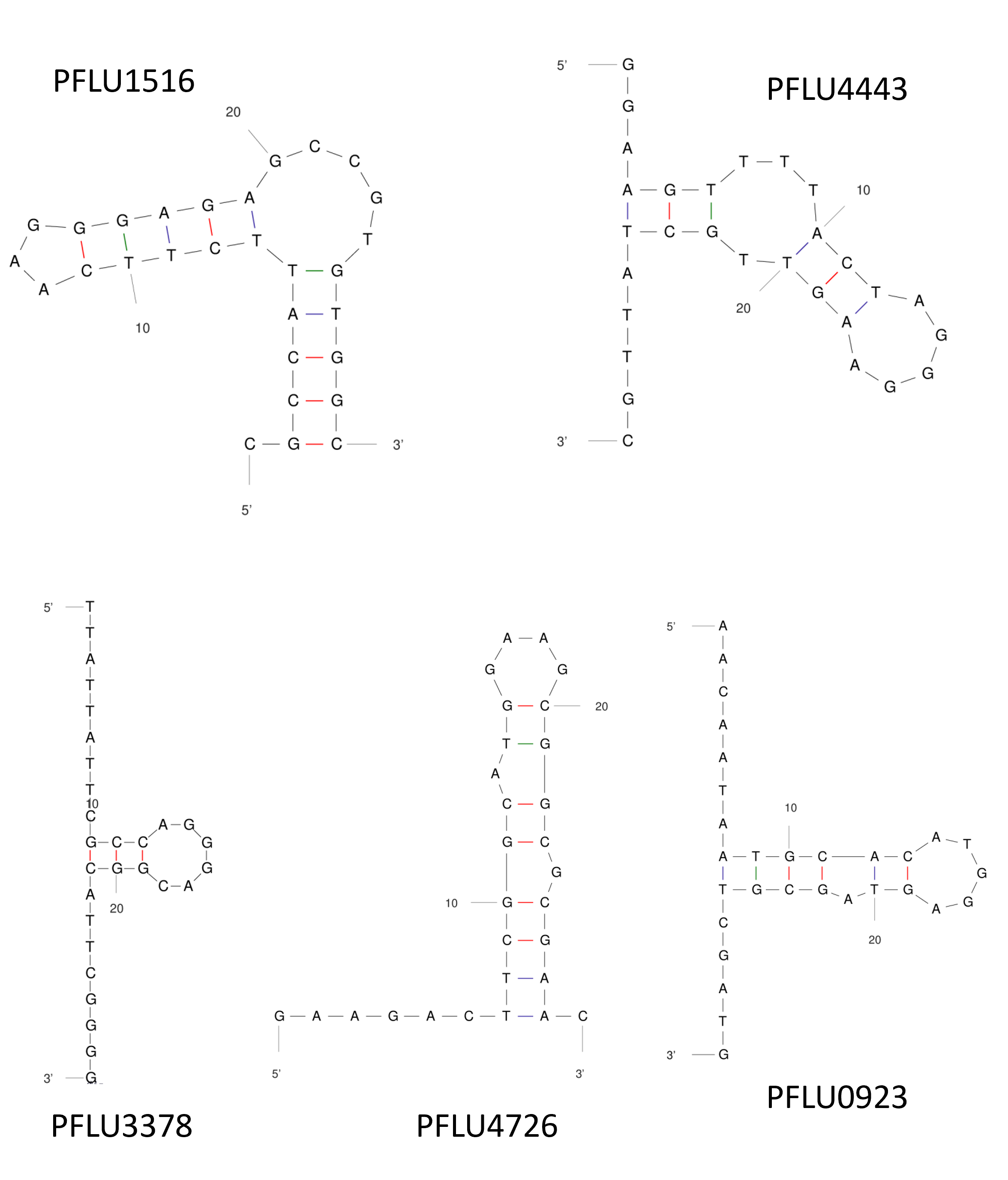

Supplement: S4 Fig — Secondary structure predictions of key oligos showing the positioning of the AnGGA binding site within the hairpin loops. Images generated using IDT oligo analyser. (TIF) [file pbio.3001988.s004.tif]

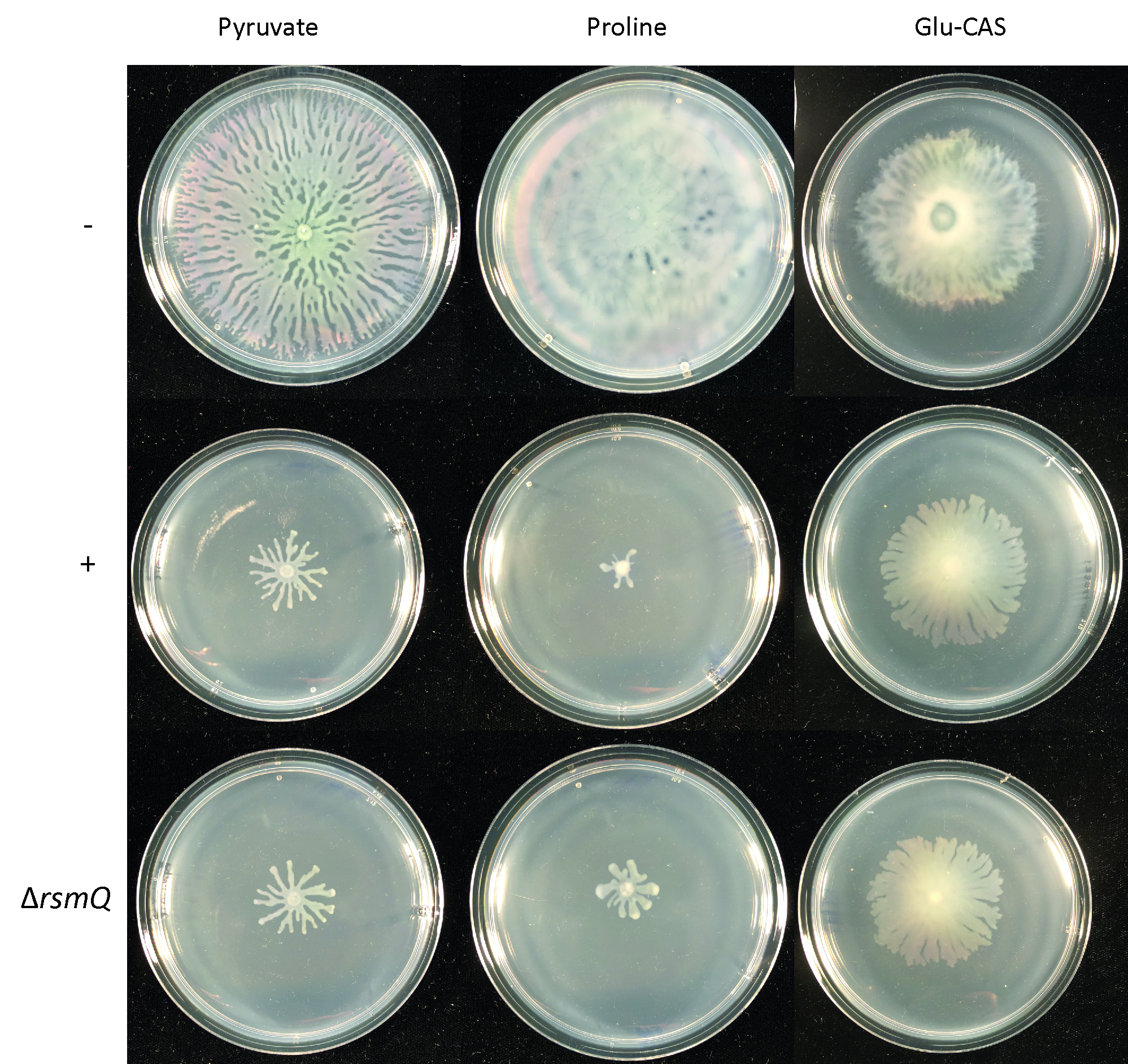

Supplement: S7 Fig — (a) Swarming motility after 72 h for SBW25 cells either plasmid free (-) or carrying pQBR103Km (+) or pQBR103Km-ΔrsmQ grown on 0.5% M9 media with the carbon source indicated. (TIF) [file pbio.3001988.s007.tif]
