# Supplementary material for: Plasmids manipulate bacterial behaviour through translational regulatory crosstalk
Source: PLoS Biol. 2023 Feb 14;21(2):e3001988. doi: 10.1371/journal.pbio.3001988 (PMC9928087; doi:10.1371/journal.pbio.3001988)
Supplement: S3 Table — (DOCX) [file pbio.3001988.s014.docx]

**Table S3** Table of Strains and Plasmids

| Strains | Description | Reference |
| --- | --- | --- |
| *P. fluorescens* | | |
| SBW25 | Environmental *P. fluorescens* isolate | 1 |
| SBW25ΩGent^r^ | SBW25 with a gentamicin resistance cassette in a neutral location within the genome | This study |
| SBW25 ΩStrep^r^-LacZ | SBW25 with a streptomycin resistance cassette and *LacZ* in a neutral location within the genome | This study |
| SBW25 ΩStrep^r^-LacZ + pQBR103^Km^ | SBW25 ΩStrep^R^-LacZ carrying pQBR103^Km^ | This study |
| sbw25 ΩStrep^r^-LacZ *+* pQBR103 ^Km^ *ΔrsmQ* | SBW25 ΩStrep^R^-LacZ carrying pQBR103^Km^ *ΔrsmQ* | This study |
| SBW25 ΩGent^r^ + pQBR103 ^Km^ | SBW25 ΩGent^R^ carrying pQBR103^Km^ | This study |
| SBW25 *Ω*Gent^r^ *+* pQBR103 ^Km^ *ΔrsmQ* | SBW25 ΩGent^R^ carrying pQBR103^Km^ *ΔrsmQ* | This study |
| SBW25ΩGent^r^ Δ*rsmA* | SBW25 ΩGent^R^ with Δ*rsmA* (PFLU4746) locus truncated to the first and last 5 AAs. | This study |
| SBW25ΩGent^r^ Δ*rsmA* + pQBR103 ^Km^ | SBW25 ΩGent^R^ Δ*rsmA* carrying pQBR103^Km^ | This study |
| SBW25ΩGent^r^ Δ*rsmA +* pQBR103 ^Km^ *ΔrsmQ* | SBW25 ΩGent^R^ Δ*rsmA* carrying pQBR103^Km^ *ΔrsmQ* | This study |
| SBW25ΩGent^r^ Δ*rsmE* | SBW25 ΩGent^R^ with Δ*rsmE* (PFLU4165) locus truncated to the first and last 5 AAs. | This study |
| SBW25ΩGent^r^ Δ*rsmE* + pQBR103 ^Km^ | SBW25 ΩGent^R^ Δ*rsmE* carrying pQBR103^Km^ | This study |
| SBW25ΩGent^r^ Δ*rsmE +* pQBR103 ^Km^ *ΔrsmQ* | SBW25 ΩGent^R^ Δ*rsmE* carrying pQBR103^Km^ *ΔrsmQ* | This study |
| SBW25ΩGent^r^ Δ*rsmI* | SBW25 ΩGent^R^ with Δ*rsmE* (PFLU4324) locus truncated to the first and last 5 AAs. | This study |
| SBW25ΩGent^r^ Δ*rsmI* + pQBR103 ^Km^ | SBW25 ΩGent^R^ Δ*rsmI* carrying pQBR103^Km^ | This study |
| SBW25ΩGent^r^ Δ*rsmI +* pQBR103 ^Km^ *ΔrsmQ* | SBW25 ΩGent^R^ Δ*rsmI* carrying pQBR103^Km^ *ΔrsmQ* | This study |
| SBW25ΩGent^r^ Δ*gacS* | SBW25 ΩGent^R^ with Δ*rsmE* (PFLU3777) locus truncated. | 2 |
| SBW25ΩGent^r^ Δ*gacS* + pQBR103 ^Km^ | SBW25 ΩGent^R^ Δ*gacS* carrying pQBR103^Km^ | This study |
| SBW25ΩGent^r^ Δ*gacS +* pQBR103 ^Km^ *ΔrsmQ* | SBW25 ΩGent^R^ Δ*gacS* carrying pQBR103^Km^ *ΔrsmQ* | This study |
| *E. Coli* | | |
| BL21-(DE3) | Sm^R^, K12 *recF143 lacI^q^ lacZΔ.M15*, *xylA* | Novagen |
| DH5α | *endA*1, *hsdR*17(r_K_-m_K_+), *supE*44, *recA*1, *gyrA* (Nal^r^), *relA*1, Δ(*lacIZYA-argF*)U169, *deoR*, Φ80*dlacΔ(lacZ)M15* | 2 |
| BTH101 | F-, cya-99, araD139, galE15, galK16, rpsL1 (Str^r^), hsdR2, mcrA1, mcrB1. | 3 |
| Plasmids | | |
| pME6032 | Tet^R^, P_K_, 9.8 kb pVS1 derived shuttle vector | 4 |
| pME6032-rsmQ | pME6032 containing the ORF for *rsmQ* between the EcorI/XhoI restriction sites | This study |
| pKNT25 | Plasmid for constructing N-terminal fusions to T25, Kan^R^ | 3 |
| pKNT25-RsmQ | pKNT25 with the ORF of *rsmQ* cloned within the EcoRI/BamHI sites | This study |
| pKNT25-RsmA | pKNT25 with the ORF of *rsmA* cloned within the EcoRI/BamHI sites | This study |
| pKNT25-RsmI | pKNT25 with the ORF of *rsmI* cloned within the XbaI site using Gibson assembly | This study |
| pKNT25-RsmE | pKNT25 with the ORF of *rsmE* cloned within the XbaI site using Gibson assembly | This study |
| pUT18c | Plasmid for constructing C-terminal fusions to T18, Carb^R^ | 3 |
| pUT18c –rsmQ | pUT18C with the ORF of *rsmQ* cloned within the EcoRI/BamHI sites | This study |
| pUT18c -rsmA | pUT18C with the ORF of *rsmA* cloned within the EcoRI/BamHI sites | This study |
| pUT18c-rsmI | pUT18C with the ORF of *rsmI* cloned within the XbaI site using Gibson assembly | This study |
| pUT18c-rsmE | pUT18C with the ORF of *rsmE* cloned within the XbaI site using Gibson assembly | This study |
| pKT25-Zip | pKT25 carrying the leucine zipper of GCN4, Km^r^ | 3 |
| pUT18-zip | pUT18 carrying the leucine zipper of GCN4, Km^r^ | 3 |
| pQBR103 *^km^* | Environmental pQBR103 plasmid with kanamycin resistance marker placed within a neutral location | This study |
| pQBR103 *^km^ ΔrsmQ* | pQBR103 ^km^ with amino acids 2-46 from the ORF of *rsmQ* removed using allelic exchange. | This study |
|  |  |  |

1. Rainey PB, Bailey MJ. Physical and genetic map of the Pseudomonas fluorescens SBW25 chromosome. Mol Microbiol. 1996;19(3):521-33. Epub 1996/02/01. PubMed PMID: 8830243.
2. E. Harrison, D. Guymer, A. J. Spiers, S. Paterson, M. A. Brockhurst, Parallel Compensatory Evolution Stabilizes Plasmids across the Parasitism-Mutualism Continuum. Curr. Biol. 25, 2034–2039 (2015).
3. Woodcock DM, Crowther PJ, Doherty J, Jefferson S, DeCruz E, Noyer-Weidner M, et al. Quantitative evaluation of Escherichia coli host strains for tolerance to cytosine methylation in plasmid and phage recombinants. Nucleic Acids Res. 1989;17(9):3469-78. PubMed PMID: 2657660.
4. Karimova G, Pidoux J, Ullmann A, Ladant D. 1998. A bacterial two-hybrid system based on a reconstituted signal transduction pathway. Proc Natl Acad Sci U S A 95:5752-5756.
5. Heeb S, Itoh Y, Nishijyo T, Schnider U, Keel C, Wade J, et al. Small, stable shuttle vectors based on the minimal pVS1 replicon for use in gram-negative, plant-associated bacteria. Molecular plant-microbe interactions : MPMI. 2000;13(2):232-7. PubMed PMID: 10659714.
