# Supplementary material for: Plasmids manipulate bacterial behaviour through translational regulatory crosstalk
Source: PLoS Biol. 2023 Feb 14;21(2):e3001988. doi: 10.1371/journal.pbio.3001988 (PMC9928087; doi:10.1371/journal.pbio.3001988)
Supplement: Attachment — Submitted filename: Response to reviewers.docx [file pbio.3001988.s029.docx]

**Reviewer #1**

We thank the reviewer for their thorough critique of our study. Below, we have tried to explain the reasoning behind our experimental approach and address specific concerns.

Major comments:

- Introduction, 3rd paragraph, lines 54-55: I don't think this statement is correct. The plasmid-located RNA-binding proteins FopA and FinO have both been reported to interact with transcripts from the main chromosome (see Gerovac et al, RNA, 2020 and El Mouali et al, NAR 2021).

We thank the reviewer for bringing these studies to our attention. We have amended the text for accuracy and to include citations to both papers. Line 54 now reads: ‘*Although specific targeted instances of translational control have been observed (14, 15), it is currently unknown whether conjugative plasmids are able to manipulate global translational regulatory pathways.’*

- Fig. 2: As pointed out above, I do not fully understand the motivation for these experiments. In vivo experiments such as RIP-Seq and CLIP-Seq would be more suitable to identify the interaction partners and binding preferences of RsmQ. Also, why did the authors decide to use ssDNA in these experiments rather than RNA?

CLIP-seq is certainly a useful technique to identify the RNA binding preferences of a target protein. While we considered this, we believe the approach we have taken to characterise RsmQ is equally valid. Our biochemical assays have enabled us to precisely define the RNA binding sequence of RsmQ independently of cellular context. Combining this data with bioinformatic analysis, RNA-seq and proteomics then allowed us to reconstruct the RsmQ regulon in detail (including both direct and indirect targets) under our test conditions. CLIP-seq reveals RNA-protein binding sites *in vivo* but says little about the consequences of binding for gene translation. Thus, global gene expression analysis (e.g. by RNA-seq/proteomics) is still necessary to understand how RNA binding translates to physiological changes in the cell.

We used ssDNA in these experiments for several reasons. ssDNA molecules (i.e. PCR primers) are significantly cheaper, more stable and much easier to source than either custom RNAs or hybrid DNA-RNA oligos. Furthermore, ssDNA is compatible with ReDCaT SPR, enabling the rapid, high throughput screening of multiple protein-oligo binding pairs. Concerning the possibility that using ssDNA rather than RNA probes may generate experimental artefacts, characterised Rsm proteins interact exclusively with A & G bases in their target sequences and not with the RNA backbone (Duss et al. (2014), *Nucleic Acids Research* **42**(8), 5332–5346). Therefore, this risk was judged to be very low. To confirm that our approach was robust, we included 2 scrambled, non-specific binding sequences in our assays to test for non-specific interactions with the probes. In response to a query from Reviewer 2, we have also tested RsmE using ReDCaT SPR with ssDNA probes and have added this binding data to the manuscript (Supplementary figure 2b and c and mentioned within the text (Lines 192-198 in tracked version)).

- Fig. 3 and lines 202-218: the large discrepancies between the transcriptomic and proteomic experiments are difficult to follow. In bacteria, translation repression is typically associated with transcript decay as the ribosomes fail to protect the mRNA from ribonucleolytic cleavage. Thus, one would expect that changes in the proteome should also be detectable at the transcript level (at least for the majority of transcripts).

Translational repression in bacteria can be accompanied by transcript decay. However, we have previously shown that this is not always the case in *P. fluorescens* SBW25 (Grenga et al (2017), *Frontiers in Microbiology* **8**, 1784). In this study we observed significant disruption of both mRNA translation and protein abundance upon *hfq* deletion that was not accompanied by corresponding changes in mRNA abundance. These new data are thus consistent with our earlier study.

While *rsmQ* deletion does not lead to significant changes in transcript abundance, carriage of pQBR103 does lead to more substantial transcriptional changes in SBW25. To provide additional context to our RNA-seq analysis we have added the dataset for plasmid-free SBW25, which enables the impact of plasmid carriage to be assessed alongside the effect of *rsmQ* (new supplementary figure S3) and discuss these new data in the relevant sections of the results.

- Lines 242-250: this paragraph is highly speculative and does not add relevant information to the paper.

We believe that evidence for RsmQ interaction with other global regulatory proteins is important and should be highlighted in the manuscript. That said, we appreciate the reviewer’s point that the paragraph as written is somewhat speculative. The text has been altered to the following (now lines 252-262 in the tracked changes version):

*‘As well as genes directly involved in specific cellular processes, we also observed alterations to the expression levels of the global regulator Hfq (PFLU0520), which was shown to be downregulated in a ΔrsmQ background, with corresponding shifts in a fraction of the published SBW25 Hfq regulon (58). Intriguingly, one of the few plasmid-encoded proteins that was significantly affected by RsmQ was an Hfq homologue (pQBR0137), whose abundance increased upon rsmQ deletion (Table S1). However, only the chromosomally encoded Hfq possesses a predicted RsmQ binding site.’*

- Fig. 5: the observation that RsmQ can form heterodimers with other CsrA-like proteins is potentially interesting, however, these experiments would be more informative if they were carried out in P. fluorescens rather than using plasmid-borne overexpression in Escherichia coli. In addition, it is not clear if and how these heterodimers would affect the RNA-ligand profiles of these proteins.

BACTH analysis to examine bacterial protein-protein interactions is typically conducted in a specially adapted *E. coli* test strain, as we have done in this experiment. Repeating these assays in *P. fluorescens* would involve a substantial amount of genetic manipulation that we did not consider to be warranted, given *E. coli* and *P. fluorescens* are both γ-proteobacterial species with similar cytosolic environments. Attempts to confirm heterodimerisation *in vitro* unfortunately proved unsuccessful, as the Rsm proteins homodimerise, have near identical molecular weights and readily cross-react to antibodies raised against each other. Given the unambiguous results of our BACTH assays and their relevance to RsmQ function in SBW25 we feel these data should remain in the paper.

As the reviewer correctly notes, the biological reason for heterodimer formation is currently unclear. It is possible that this functions to suppress host Rsm function, or to enable binding to different mRNA binding sequences. Following up this initial finding is an interesting and important challenge; however, we feel this major undertaking is beyond the scope of the current study.

- Figs. 8b and c: I do not think these results are informative given the large variation among the results.

*[We think the reviewer may be referring to Figs. 7b and c here.]* The variation seen in these assays is not particularly unusual as each datapoint represents a biologically independent replicate of a highly complex process. Despite the high variation seen, our results are both repeatable and (in 7c) significant. For previously published examples please see: Harrison et al. (2015), *Current Biology* **25**(15), 2034-9 and Hall et al (2020), *Front Micro.* **11**, 1846.

Minor comments:

- Lines 108-109: given that the plasmid encodes an CsrA-like protein, i.e. RsmQ, it would be interesting to know if the plasmid also encodes homologues of the RsmY and RsmZ sRNAs.

This is an excellent suggestion. We examined the pQBR103 sequence for sRNA motifs, but did not find anything. Given that RNA-seq has been used in the past to detect sRNAs encoded in bacterial chromosomes, we also examined our RNA-seq datasets for signs of ncRNA transcription. However, we could not detect anything resembling RsmY/Z. This does not exclude the presence of uncharacterised sRNAs that interfere with host function, which would be an intriguing area for future study.

- Fig. 6b: I think it would be useful to quantify the degree of swarming observed in these assays.

We agree with the reviewer. For rigor, we repeated this experiment again with a further 4 biological replicates (3 technical replicates). We used ImageJ analysis to quantify the swarm diameters achieved in our assays and have added this data to new figure S5.

- Fig. 7c: please clarify why conjugation rates are significantly higher in M9Pyr when compared to KB and M9Glc.

Conjugation is a tightly controlled process within bacterial cells. Previous work has found that environmental cues and nutrient environments can have an impact on conjugation rate, or at least on the derepression of *tra* genes (Koraimann & Wagner, 2014). There has been very limited work examining why conjugation rate may vary under different nutrient conditions, but differential conjugation rates have been seen between rich media (KB) and potting soils (Hall et al, 2015) as well as when compared in a variety of food substances (Madrie et al, 2009). So, whilst we observed higher conjugation rate, and this is almost certainly due to cues from the nutrient environment, or indeed quorum sensing cues, this is something that remains poorly understood within the field. We have added a line in the discussion at line 503 in the tracked changes version mentioning this.

- Please add error bars to Fig. 8a.

The screening experiment in Fig 8a was conducted only once, hence the lack of error bars. In response to comments from the other reviewers we performed additional BioLog plates and repeated these assays several more times to further support the growth bistability effect of Δ*rsmQ* on different media. The full set of BioLog assay data for selected carbon sources is now included as Figure 8a depicting difference compared to plasmid free to make the differences observed easier to comprehend for the reader. All carbon source data are available in both Figure S10 and Table S2. Interestingly, expanding our growth rate experiments suggested that the observed bistability phenotype may not be plasmid-specific as initially suspected. However, these assays did lead us to uncover definitive plasmid effects when it came to carbon source utilisation. This has been updated in the text (Line 410 onwards) and Figure 8.

**Reviewer #2:**

We thank the reviewer for their positive and insightful assessment of our manuscript. Specific responses to their concerns are outlined below.

Specific comments are as follows:

- Considering RsmQ has a similar binding site as compared to other RsmA-like, and that eventually it has common targets with endogenous Rsm, could the authors assess what would be the "preferred" repressor for some of the common targets. In other words what is the affinity of the different regulators for their RNA targets. This is an important question since it may address whether RsmQ interferes with host gene expression by competing with residents Rsm. The same question holds true for the concept where RsmQ binds RsmYZ. Is the affinity different as compared to the endogenous RsmA-like?

This is an insightful point by the reviewer. To investigate this further we purified SBW25 RsmA and RsmE and examined their affinities for the oligos tested against RsmQ. Unfortunately, RsmA bound non-specifically to the SPR chip, meaning we could not produce data for this protein. Conversely, RsmE bound specifically to oligonucleotides with the predicted AnGGA binding site. Interestingly however, RsmE binding was both weaker and took place across a more restricted range of sequences than with RsmQ. These data have been added to Supplementary figure S2b and c and mentioned within the text (lines 192-198 in tracked version). This result is consistent with RsmQ outcompeting RsmE for RNA binding sites, and we mention this in the text. We further tested RsmE against both RsmY/Z and the upstream regions of the RsmQ target genes, however no binding was observed (%R_max_ <0), we therefore did not include this data.

- This is minor but do the authors have any clues on what the source for pQBR103 is in the environment. It seems from their genomic analysis that RsmQ-encoding plasmids are mostly found in Legionellaceae and Pseudomonadaceae. Is there any physiological explanation for this bias?

pQBR103 was originally isolated with a large number of other similar plasmids in the 1990s (Lilley and Bailey (1997), *Appl. Env. Micro.* **63**, 1577–1583). It appears to naturally occur within *Pseudomonas* populations within the soil and other natural environments. Regarding the high number of RsmQ proteins associated with *Pseudomonadaceae* and *Legionellaceae* plasmids, we think this is an artefact of the predominance of these plasmids in the COMPASS database. The revised Figure 1a (where absolute values are replaced with relative proportions) shows a more even distribution of RsmQ-like proteins across plasmids from different genera.

- In a growth experiment does RsmQ and the other endogenous Rsm expressed simultaneously? Can they be expressed at distinct stages which would contribute to provide them with alternative and not competitive control on target RNAs.

This is an interesting question. To test this, we extracted RNA samples from SBW25 ± pQBR103 at different time points throughout the cell cycle and used qRT-PCR to measure the abundance of *rsmQ, rsmA* and *rsmE* transcripts, relative to a *rpoS* control. These data have been added to Figure S11, with a description of the results on Lines 319-323. In short, no real differences were observed between the expression levels of each protein at any stage of the growth cycle we examined, with *rsmA* (Figure S11a) and *rsmI* (Figure S11d) showing little-to-no expression between mid-log and early stationary phase. Interestingly however, *rsmE* (Figure S12c) was expressed at a high level (greater than *rpoS*) and expression levels decreased over time. This decrease was seen to be significant, with a Two-way ANOVA showing no effect of plasmid carriage, but a significant effect of time (p<0.001). A similar observation was made for *rsmQ*, which was observed to be expressed at all growth phases with a decrease in abundance observed over time, with abundance being significantly different over time (One-way ANOVA, p<0.01 with mid-log and late-log being significantly different to early stationary phase expression). Taken together, this would suggest that RsmQ and RsmE are direct competitors, but that RsmA and RsmI are not.

- It is noted that RsmQ will trigger biofilm formation and thus increases conjugative opportunities. Yet the endogenous Rsm would rather contribute to prevent biofilm formation. It is hard to reconcile this antagonism when comparing regulators which essentially have the same targets. For example, if RsmQ sequesters RsmYZ then there will be more freed RsmA and thus more repression in biofilm. I may have missed some interpretation there, but it will be worth discussing this point in more clear details.

While RsmA has been shown to suppress biofilm formation in other *Pseudomonas* spp., this does not appear to be a universal trait. When we tested the impact of *rsm* gene deletion on SBW25 behaviour (New supplementary figure S7), whilst there is still a clear effect of plasmid carriage on biofilm formation, we did not see significant effect of *rsm* deletion on biofilm formation for any of the chromosomally (or plasmid encoded) Rsm proteins. This is consistent with previous published work on the Gac/Rsm system (see: Lapouge *et al.* (2008), *Mol. Micro.* **67**(2), 241-253). Interestingly, the deletion of *gacS* leads to an increase in biofilm formation, with similar levels of observed for plasmid free and pQBR103 ±*rsmQ* in this strain. This may suggest either redundancy within the Rsm system or that there is another mechanism by which biofilm formation is controlled. We have tweaked the language throughout the paper to clarify the relationship between Rsm proteins and biofilm formation in SBW25.

- The inconsistencies regarding growth in different biological replicates explained by RsmQ involved in a bistable metabolic system is appealing but is rather preliminary and would need further support (Lines 377-382 and lines 502-508).

We agree that this finding could be better supported. To investigate further, we ran an additional 2 BioLog growth assays in parallel to assess the magnitude and extent of metabolic bistability associated with pQBR103 and RsmQ. These data are included in Figure 8a, S10 and table S2. While these new data are largely consistent with our earlier results, for some carbon sources natural variation appears to be a simpler explanation for the data than plasmid-induced metabolic bistability. As our new data does not provide strong additional support for bistability in different carbon sources we have altered figure 8 to reflect this, focusing instead on definitive growth differences observed upon plasmid carriage in different carbon sources. We have amended the results and discussion (at Lines 395 and 589) to reflect this.

- It seems that the presence/absence of the plasmids compares quite differently when looking at plasmids with or without a functional RsmQ (Lines 312-316). That would mean that there are additional regulatory elements on the plasmid that may influence host response. This is not really discussed and deserved to be.

This is a very good point. pQBR103 contains several additional regulatory genes that control host ecological traits. These include transcriptional regulators, translational regulators like the *hfq* homolog we mention on lines 242-250, as well as second messenger signalling proteins. Several of these proteins are under active investigation in our lab and will be the subject of upcoming papers. We have revised the discussion to consider the possible impact of additional regulatory elements in more detail (Line 550 onwards).

**Reviewer #3:**

We thank the reviewer for their positive and detailed review of our manuscript. Their specific concerns are addressed below:

Specific Comments:

- L60: 'Rsms'. Plural protein names may be confusing. Please consider rephrasing. A suggestion could be 'rsm genes encode small proteins…'

This has been rephrased in the text (line 60) to: *‘Rsm proteins are able to interact directly with the bases AnGGA around the ribosome binding site (RBS) of their target transcript (27–30).’*

- L113. Please clarify which criteria was followed to identify rsmQ homologs. Percentage of homology and identity?

The methodology for identifying rsmQ homologs is described on lines 845-848, and has been edited slightly for clarity: *‘The COMPASS database (40) was downloaded from* [*https://github.com/itsmeludo/COMPASS*](https://github.com/itsmeludo/COMPASS) *and annotated using PROKKA 1.14.6 (93) using the default settings* *to identify plasmid-borne rsmQ/csrA genes. PROKKA uses BLAST+ matches with the curated UniProtKB (SwissProt) databases to annotate proteins, followed by hidden Markov model (HMM)-based searches.’*

- Fig 1. It seems that in fig 1a absolute numbers are presented. It would be more informative to include the percentage of plasmids, as the total number of rsmA homologs will likely depend on the number of plasmids per genus in the database.

As requested, we have updated Figure 1a to show the percentage of plasmids rather than the absolute values. However, we think that the absolute numbers also provide valuable information, so we have added the original figure to the supplementary information (Figure S1c).

- Fig 1C. The figure legend is confusing. It should state somewhere that all these belong to the Pseudomonas genus. Also, I wonder if the authors found rsmA homologs with mutations and/or deletions that could potentially lead to a loss-of-function phenotype. In that case, could be a hint pointing to compensatory evolution.

We have amended the text in Figure 1c to include the genus of each species (all *Pseudomonas* apart from the indicated *E. coli*). The way in which the annotation is performed means that we are looking for similar ORFs, and therefore information about compensatory mutations resulting from deletions is likely to be missed. That said, all of the ORFs we have looked at in detail, (which is by no means all of them) appear functional, i.e. we see no significant changes to predicted structure or presence of the VHRE/D motif.

- L117 states that 'over 50% of Legionellaceae (18/30) plasmids contained rsm homologues', but in Fig1a, there seem to be more than 20. Also, the text states 41 plasmids for Pseudomonas, but the figure seems to indicate way more. Please clarify.

This apparent inconsistency occurs because the number of homologs (indicated within the figure) in *Legionellaceae* is >1 on some plasmids. Therefore, the number of homologs present are greater than the number of plasmids. With the change of figure 1a to a proportion of plasmids rather than a numerical value this should reduce this confusion as the values should now be consistent. Lines 118-120 have been updated in line with the new figure.

-Fig S1. Is there a reason why there is no distribution for Rhodanobactereaceae and Thermomicrobiacea? I guess it is because there is only one plasmid per family in the database. However, from L1127 I understood that data represented corresponds to families 'with >20 plasmids and ≥1 plasmid-encoded CsrA/RsmA homologue.' Please clarify.

We have updated this figure and legend in the supplementary information for clarity.

- L179: For clarity, please explain here why those mutants are expected to affect the conserved VHRD/E motif. Are they directly altering the motif sequence?

We have clarified this point in the text (Line 183): *‘Next, we tested if the RNA binding interaction was co-ordinated by the conserved VHRD/E motif at the C-terminus of RsmQ by examining the binding of two RsmQ mutants, in which key residues within the motif were changed to alanine residues (H43A and R44A), to the ssDNA probes.’*

- L181: Statistical analyses should be performed to sustain this claim. Also, there seem to be tiny error bars associated with the Rmax measures. What do they represent? Please specify in the figure legend.

This has been clarified in the text with the information about the statistical analysis performed (Two-way ANOVA with multiple comparisons) added to the figure legend. (Line 1154).

- Table S1: There are no statistical analyses of the RNA-seq and proteomic data. Without statistics is difficult to judge the significance of the results. Additionally, gene names should be standardized between the main text and table S1 (e.g., PFLU0610 vs. PFLU_0610)

Statistical analysis was performed but we agree that the reporting of this can be improved; statistical outputs have been added to the tables in S1 for both datasets and gene names have been standardised between the main text and table S1 as requested. Comprehensive data sets and full statistical analyses are available on the appropriate databases with the full set of proteomics data available on ProteomeXchange (PXD033640) and the RNAseq data is available at ArrayExpress (E-MTAB-11868).

- Figure 4. At 100 nM, the %Rmax of Hairpin ACGGA and GGA seem to slightly differ from those in fig1. Is this expected? Five of the ~25 oligos tested reach the 50% threshold, but most only at really high RsmQ concentrations (i.e., above 250-500 nM). Can the authors elaborate on why they believe this is physiologically relevant?

Figures 2 and 4 were produced 12 months apart using independent protein preparations. Minor differences in R_max_ are therefore to be expected as this is based on protein binding efficacy, which can change on a prep-to-prep basis. When overlaid, the SPR sensorgrams for the two independent assays are almost identical. This has been caveated in the text on lines 271-273 in the tracked document.

While only a few UTR sequences bound strongly to RsmQ in our assays, this is not too surprising given that these sequences were taken out of cellular context, with only a short oligo sequence tested in each case. These results should be considered as the minimal RsmQ-binding regulon rather than a complete assessment of the ability of RsmQ to bind to all tested targets. We have amended the text slightly to reflect this (line 282-3). Nonetheless, we were encouraged that most of the oligos that bound well to RsmQ had canonical AnGGA sites, while none of the negative control oligos bound at all.

- Nanomolar is typically abbreviated as nM, not 'nm'.

This has been corrected throughout.

- L263-266: The secondary structure predictions are not shown. I think it could be helpful to the reader to show them as supplementary figure.

We have added this in as a supplementary figure (Supplementary Figure S4).

- L272: It would have been nice to include a chromosomal Rsm protein in Figure 2 assays as a control. I do not think it is imperative for acceptance as it has been demonstrated elsewhere. However, it would be very appropriate to control that the binding kinetics are similar using the same experimental procedure. It could also help to put in perspective the protein concentrations used in these experiments.

Reviewer 2 raised a similar point, and we agree that these data would add valuable context to our analysis of RsmQ-oligo interactions. Data for RsmE binding to our oligonucleotide library is now included in Figure S2, as mentioned above.

- L275: A reference should be added here, and the meaning of ncRNA should be stated the first time the term is mentioned.

This has been amended in the text (Line 292) and references added.

- Fig 5a: I think this figure would benefit from showing whether each of the ssDNA oligos contains (or not) the AnGGA or GGA motif. Again, error bars are shown but not described, and there is no statistical analysis of the results.

This has been clarified within the figure legend and * symbols have been added to the graph to indicate oligos containing GGA binding sites. It is also often not reported for these kinds of experiments as Rmax values are an indicator of both binding and stoichiometry, so it is not necessarily appropriate to compare them statistically. There are other ways in which these comparisons can be done, for example looking at differences in dissociation constants, or the rate at which the protein is removed from the ligand, however for a general audience we felt that Rmax was the easiest to comprehend. Having said that, I don’t feel comfortable assigning statistical values to the differences, as it is more a case of “bound” or “not bound” than levels of binding, and the small error you get from these experiments can lead to inflated p values that are not meaningful.

- L293: a sentence briefly explaining the BATCH system may help the reader understand this experiment.

We have added the sentence below and the original reference for BATCH analysis (Lines 310-312 in the tracked changes version): *‘The BATCH system allows for the screening of interacting protein partners through a cAMP cascade activated by bringing together the two adenylate cyclase domains fused to the proteins of interest (62).’*

- L321: It is really hard to see how the loss of rsmQ restores swarming motility in this experiment. I barely see any difference between rsmQ+ and rsmQ- plates. Similarly, in figure 6B, it is clear that the presence of pQBR103 affects swarming motility, but the deletion of rsmQ barely restores swarming. This indicates that other plasmid traits may be responsible for the swarming inhibition phenotype. The authors should acknowledge this fact and discuss how it may affect their results and interpretation.

Figure 6a (Line 321): only the bottom two plates show the effect of expressing *rsmQ in trans* on motility – the top two plates contain no IPTG and hence we see no *rsmQ* expression and no effect on swarming. In response to Reviewer 1, we have quantified the swarming motility results as swarm diameters and added these results as graphs in the supplementary information (new figure S5). There is a significant effect of *rsmQ* deletion on swarming in KB media, although we agree with the reviewer that this is largely ameliorated for cells M9 Glucose Cas.

As mentioned in the response to Reviewer 2, we have reworked the discussion (Line 550 onwards) to highlight other regulatory elements that may contribute to pQBR103 control of SBW25 behaviour.

- As the only difference is the presence of rsmQ, Fig 6C could be simplified by changing the plasmid names (pME6032 and prsmQ) to pME6032 and pME6032-rsmQ.

We have changed terminology from prsmQ to pME6032-rsmQ throughout.

- Figure 7c. The number of technical and biological replicates should be indicated.

This has been clarified in the figure legend: *‘All assays were performed with 10 biological and two technical replicates. Data containing a conjugation rate count of 0 were excluded from the dataset.’*

- L341-343. Please provide statistical support for this claim.

Updated statistical analysis has been performed comparing both pQBR103^Km^ and pQBR103^Km^ -Δ*rsmQ* to a plasmid free data set (average relative fitness of WT:WT is always 1) . The significance values are added in figure 7b, and statistical analysis is discussed in the figure legend.

- L349-350. This sentence provides much-needed context for the carbon source experiments in figures 7 and S3. Please consider providing this information to the reader before moving the experiments to different carbon sources.

We have moved these two sentences to the previous paragraph which now reads (with the moved sentences highlighted in bold):

“Next, we tested whether the phenotypes influenced by *rsmQ* had an impact on the ability of SBW25 to colonise the plant rhizosphere. **The rhizosphere is a complex, heterogenous environment, with diverse nutrient sources and physical habitats. Successful colonisation involves the coordinated deployment of several distinct ecological traits.** A series of competitive wheat colonisation assays were performed between plasmid free SBW25 cells and either SBW25 plasmid free, SBW25 + pQBR103^Km^ or SBW25 + pQBR103^Km^-Δ*rsmQ*. After seven days, significantly fewer colony forming units were recovered for both plasmid carrying strains compared with the plasmid free competitor (Figure 7a). However, no significant difference was seen between strains with or without *rsmQ*.”

- L377-382. The biphasic effect warrants more investigation. It makes me wonder whether the results of the BioLog assays are also subject to a stochastic behaviour and, therefore the conclusions drawn from the experiment (L358-370). Would selecting a different colony change the observed results?

This is an excellent point. The BioLog plates were used as an initial screening approach, however we have done extensive follow up work with multiple carbon sources (only two of which are highlighted here). The biphasic effect appeared to be particularly prevalent within these two carbon sources (malate and itaconate) but not exclusive to them. To investigate this further we performed two more replicates of the BioLog plates to see if choosing a different colony would indeed show a different biphasic effect. However, what we saw was that there is a high degree of naturally occurring variation within some carbon sources (including previously examined itaconate). As we were unable to increase the robustness of our initial observations, we decided instead to highlight in the manuscript those carbon sources that showed a strong, but consistent plasmid or *rsmQ* dependent effect. We have amended Figure 8 and the text to incorporate these new findings.

- The discussion section is overly too long. Please reduce its extension thoroughly by eliminating redundancy with the results section.

The discussion section has been revised to remove redundancy and reduce its length.

- L409. PROKKA database? COMPASS instead?

This has been changed within the text.

- The authors only found an effect on conjugation rate for one out 6 conditions tested (3 carbon sources in liquid and solid conditions). I think this is a bit too anecdotal to draw general conclusions like the ones in lines 435-443. Please tone down and add a bit of nuance.

We have amended the text as requested to qualify our conclusions here.
